# Supplementary material for: Phosphatidylinositol-3 kinase signaling controls survival and stemness of hematopoietic stem and progenitor cells
Source: Oncogene. 2021 Mar 13;40(15):2741–55. doi: 10.1038/s41388-021-01733-5 (PMC8049872; doi:10.1038/s41388-021-01733-5)
Supplement: Supplementary file 1 — Supplemental Material [file 41388_2021_1733_MOESM1_ESM.pdf]

Supplemental Material

Supplemental Tables

Table S1. Marker genes used for identification of clusters in single cell RNA seq. ENSDARG numbers and gene names are given.

Table S2. Upregulated genes for scRNA-seq datasets for 36 hpf and 5 dpf per cluster. Related to Figure 5-7. Genes with a $p<0.01$ and fold change >1 are listed per cluster in a decreasing order. See associated excel file.

Supplemental Movies

Movie S1. Disintegrating HSPCs are observed in *ptena*$^{-/-}$*ptenb*$^{-/-}$ during EHT. Related to Figure 1. Four-dimensional imaging of *tg(kdrl:eGFP) ptena*$^{-/-}$*ptenb*$^{-/-}$ mutant embryo between 35 hpf and 48 hpf. HSPCs undergoing EHT. Confocal image z-stacks (2µm step size, with 40x objective and 2x zoom; anterior to the left; maximum projections of a representative embryo; time in hh:mm). HSPCs were tracked over time; time of cell tracks is color-coded (bottom right)

Movie S2. Inhibition of PI3K rescued EHT in *ptena*$^{-/-}$*ptenb*$^{-/-}$ mutant embryos. Related to Figure 3. Four-dimensional imaging of *tg(kdrl:eGFP)* transgenic *ptena*$^{-/-}$*ptenb*$^{-/-}$ mutant embryos between 35 hpf and 48 hpf following treatment with 5 µM LY294002 from 32 hpf onwards. Confocal image z-stacks (2µm step size, with 40x objective and 1x zoom; anterior to the left; maximum projections of a representative embryo; time in hh:mm). HSPCs were tracked over time; time of cell tracks is color-coded (bottom right)

Movie S3. Disintegrating HPSCs are observed in LY294002-treated wild type embryos during EHT. Related to Figure 3. Four-dimensional imaging of *tg(kdrl:eGFP)* LY294002-treated embryos between 35 hpf and 48 hpf. Embryos were treated with 5 µM LY294002 from 32 hpf onwards. HPSCs undergoing EHT. Confocal image z-stacks (2µm step size, with 40x objective and 1x zoom; anterior to the left; maximum projections of a representative embyro; time in hh:mm). HSPCs were tracked over time and time of cell tracks is color-coded (bottom right); scale bar, 40 µm.

Supplemental Figures

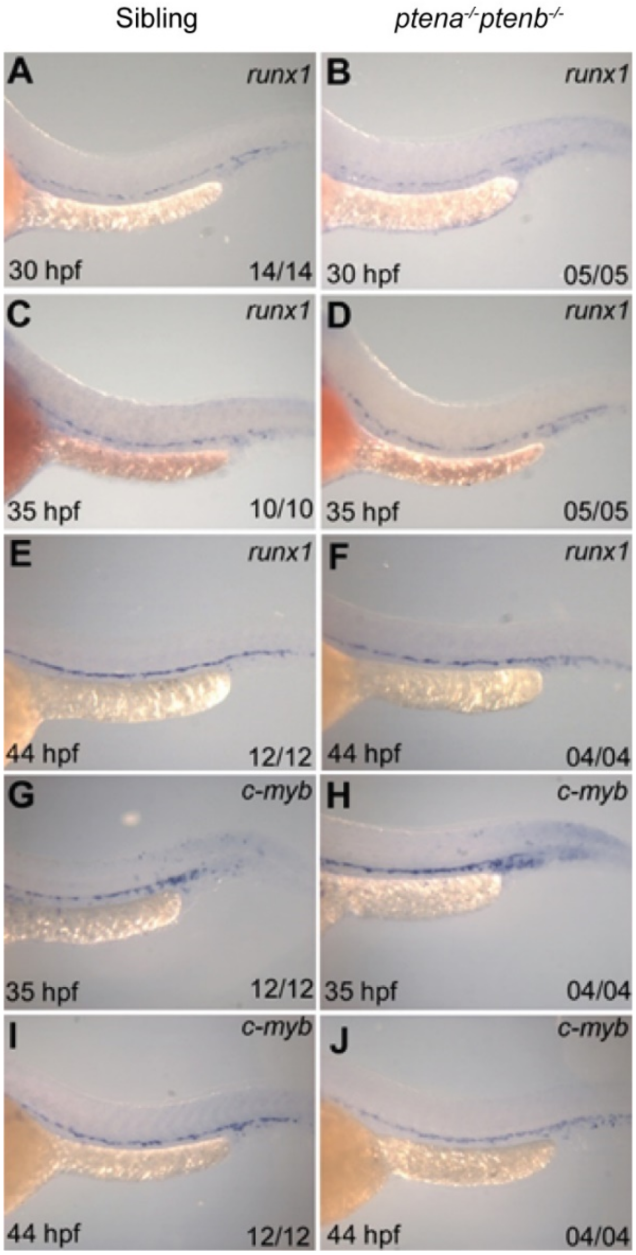

Figure S1. Hemogenic endothelium markers are present in *ptena*$^{-/-}$*ptenb*$^{-/-}$ mutants during onset of definitive hematopoiesis. Related to figure 1. *Ptena*$^{+/-}$*ptenb*$^{-/-}$ fish were incrossed and embryos were fixed at different time points as indicated (30, 35 and 44 hpf). *In situ* hybridization using HSPC markers *runx1* (a-f) and *c-myb* (g-j) was done, pictures were taken and subsequently the genotypes of these embryos was established by sequencing. No differences were observed between *ptena*$^{-/-}$*ptenb*$^{-/-}$ mutant embryos and siblings. Representative embryos are depicted with anterior to the left; the number of embryos that showed a particular pattern/total number of embryos is indicated in the bottom right corner of the each panel.

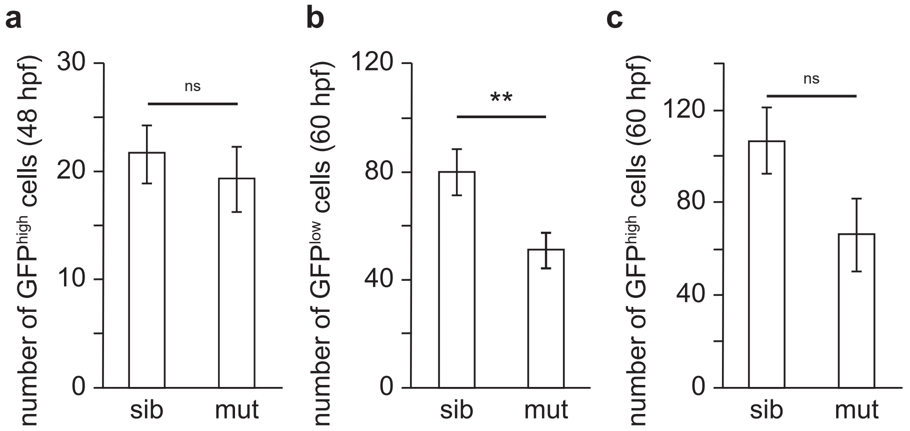

Figure S2. Reduced number of GFP^{low}, but not GFP^{high} cells in the CHT of *tg(cd41:eGFP) ptena$^{-/-}$ptenb$^{-/-}$* mutant embryos, compared to siblings. Related to figure 2. (a) the number of GFP^{high} thrombocytes at 48 hpf in the CHT of *tg(cd41:eGFP)* siblings (sib) and *ptena$^{-/-}$ptenb$^{-/-}$* mutants (mut), expressed as average number of cells. (b,c) the number of GFP^{low} HSPCs (b) and GFP^{high} thrombocytes (c) in the CHT of 60 hpf in the CHT of *tg(cd41:eGFP)* siblings (sib) and *ptena$^{-/-}$ptenb$^{-/-}$* mutants (mut), expressed as average number of cells. Note that the difer-ence in GFP^{low} HSPCs is smaller due to enhanced proliferation and the apparent difference in GFP^{high} cells is almost significant, due to an arrest in differentiation. Error bars indicate stan-dard error of the mean (SEM). Shapiro Wilk test for normal distribution and two-tailed t-test were used for statistical analysis; p-values are: (a) 0.57 (not significant, ns), (b) 0.013 (**), (c) 0.066 (not significant, ns).

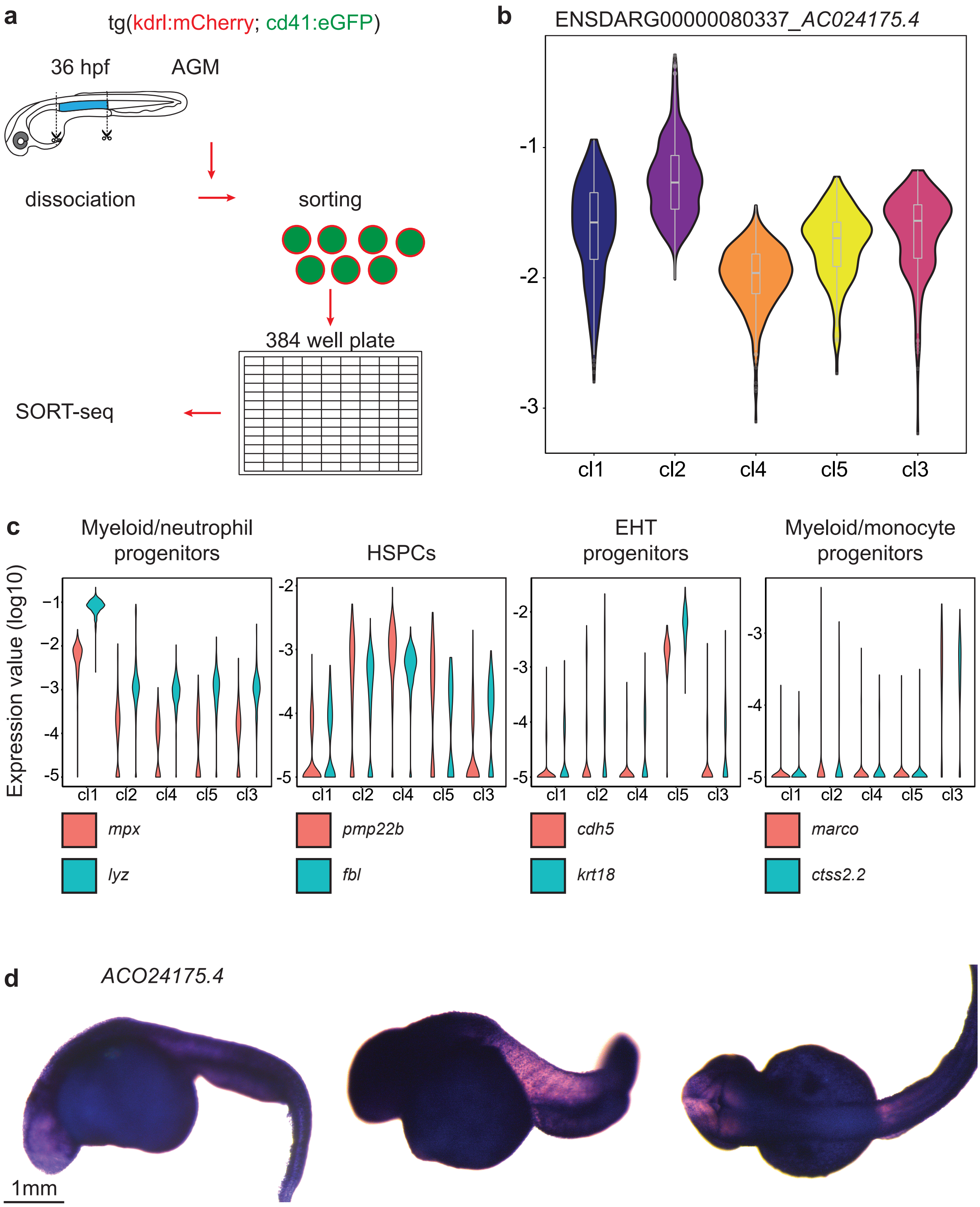

Figure S3. Single cell RNA seq of control and PI3K inhibitor treated embryos at the onset of definitive hematopoiesis. Related to Figure 5. (a) Workflow of scRNA seq. Tissue from control and LY294002-treated embryos (~2,000 each) was dissected, the AGM regions pooled, dissociated and FACS sorted, after which the SORT-seq protocol was performed. (b) Normalized expression of ENSDARG00000080337_ACO24175.4 and tmed1b over all clusters. Normalized expression is plotted on log10 scale using violin plots and boxplots. cl1: Myeloid/-neutrophil progenitor, cl2: HSPC II, cl4: HSPC I, cl5: EHT progenitor, cl3: myeloid/monocyte progenitor. (c) Normalized expression of signature genes for cluster identities using violin plots. Normalized expression value is plotted on a log10 scale. (d) whole mount ISH of 36 hpf wild type embryos using a probe specific for ENSDARG00000080337_ACO24175.4. Forward primer: 5'TTAAAGCCCCGAATCCAGGT 3', reverse primer with T7 promoter: GAGTAATAC-GACTCACTATAGGTTTTGGTAAACAGGCGAGGC. At this stage, this gene is expressed throughout the embryo at a very high level, which does not allow to distinguish between individual blood cells.

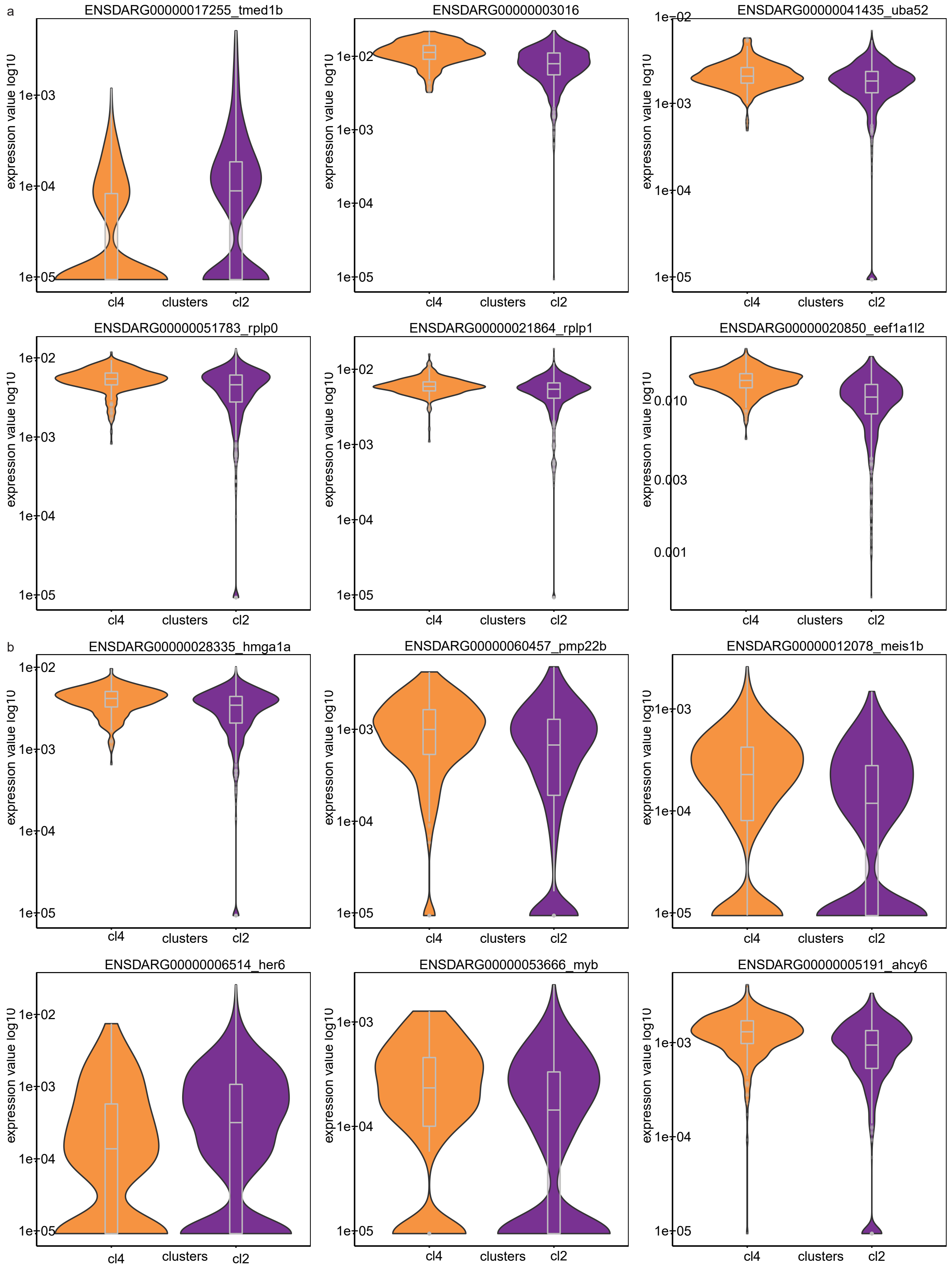

Figures S4. Cluster identities at 36 hpf for LY294002-treated embryos and their controls for only HSPCs clusters. Related to figure 5. Normalized expression of signature genes for HSPCs II (a) and HSPCs I (b) using violin plots. Normalized expresssion value is plotted on a log10 scale. Expression of all genes is significantly different between clusters (t-test, $p<0.001$). Cluster 4 is HSPCs I, cluster 2 is HSPCs II.

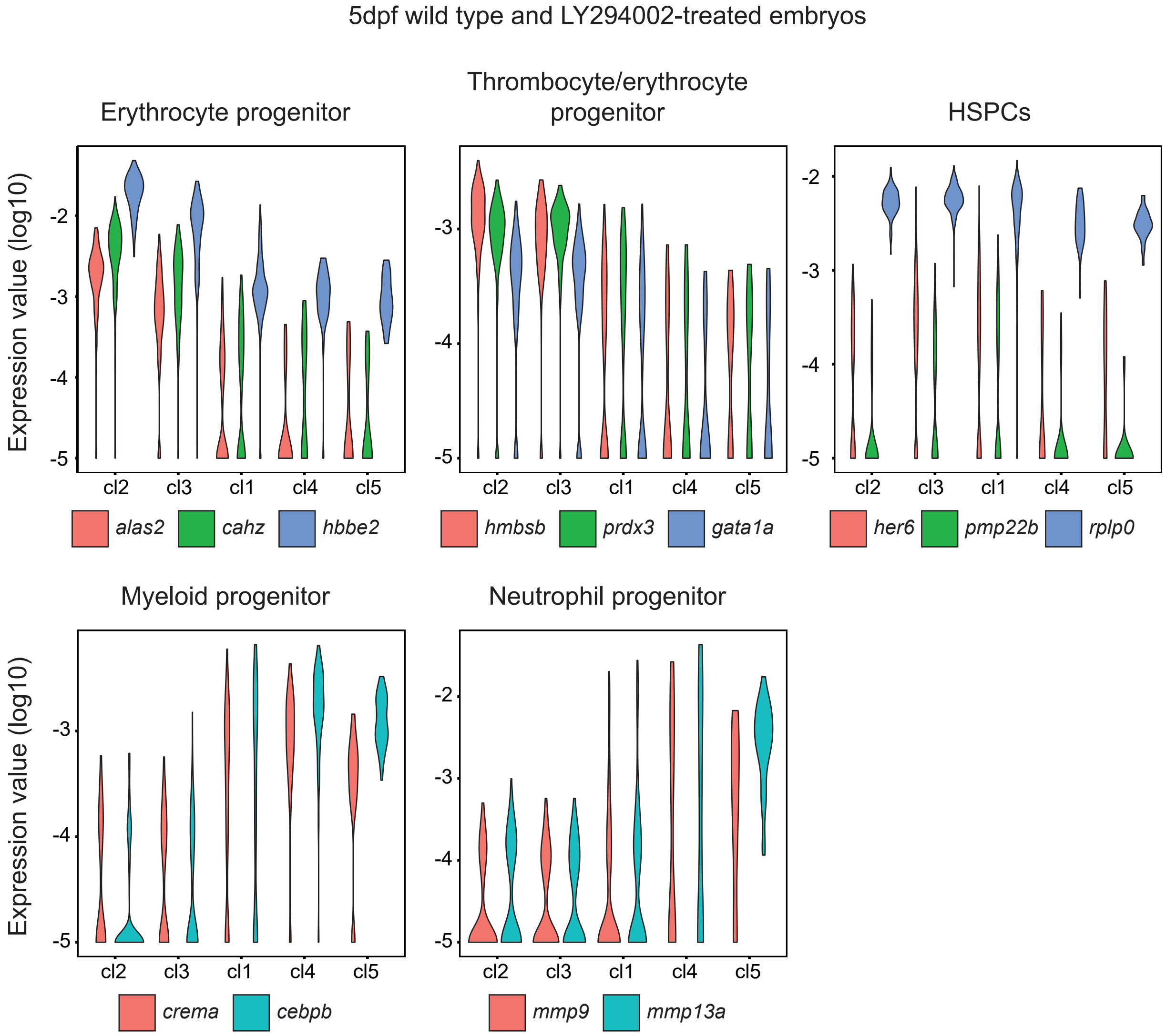

Figure S5. Cluster identities at 5 dpf for wild type and LY294002-treated embryos. Related to Figure 6. Normalized expression of signature genes for cluster identities using violin plots. Normalized expression value is plotted on a log10 scale.

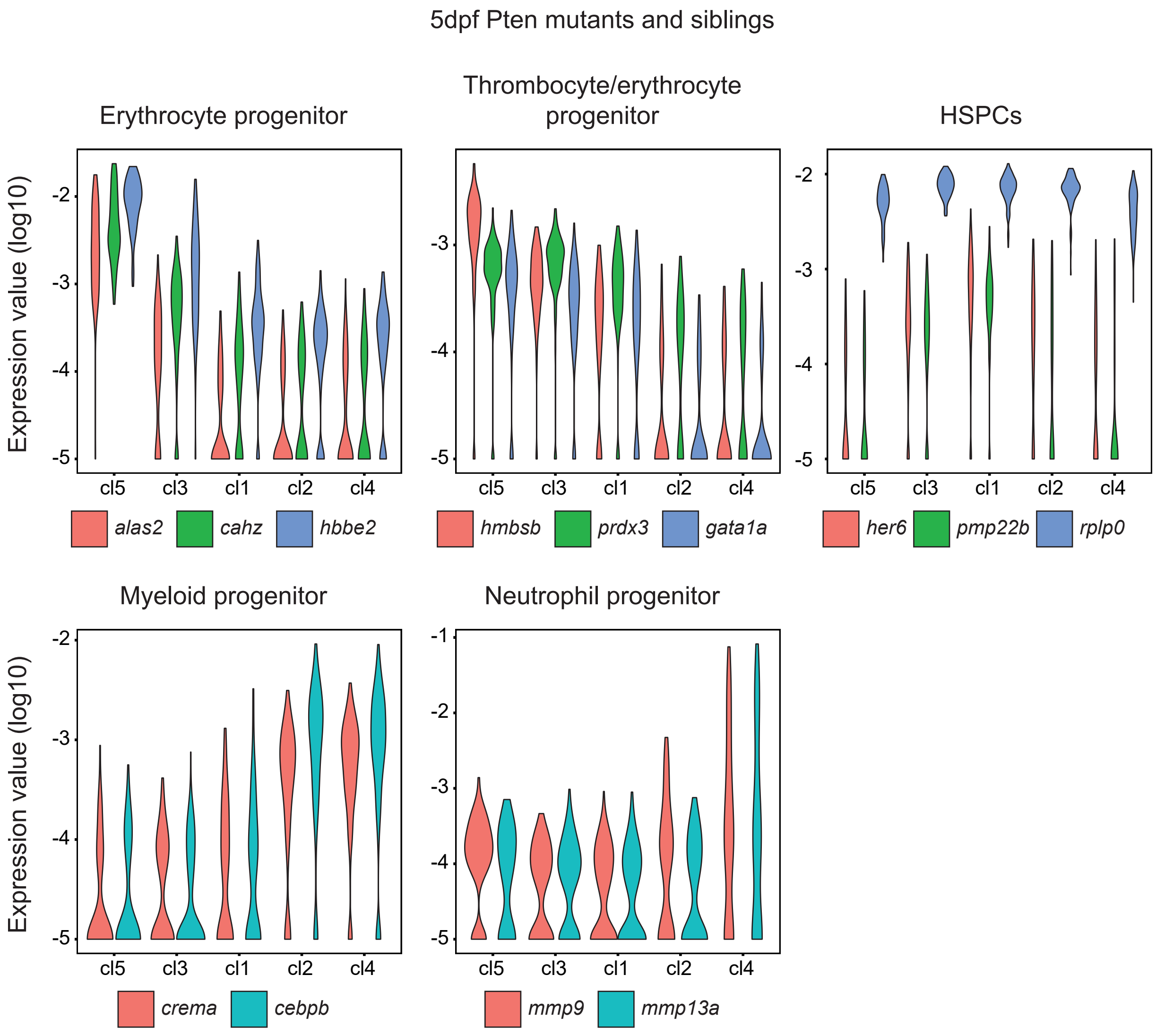

Figure S6. Cluster identities at 5 dpf for ptena-/-ptenb-/- mutant embryos and their siblings. Related to Figure 7. Normalized expression of signature genes for cluster identities using violin plots. Normalized expression value is plotted on a log10 scale.
